# The Ammonia Oxidising Archaeon *Nitrosopumilus maritimus* Does Not Alter Iodine Oxidation State in Oxic Seawater

**DOI:** 10.1111/1758-2229.70168

**Published:** 2025-08-10

**Authors:** Alison L. Webb, Barbora Oudova‐Rivera, Martyn Ward, Lucy J. Carpenter, Laura E. Lehtovirta‐Morley, Rosie Chance

**Affiliations:** ^1^ Wolfson Atmospheric Chemistry Laboratories University of York York UK; ^2^ School of Biological Sciences University of East Anglia Norwich UK

**Keywords:** ammonium, archaea, iodate, iodide, nitrification, *Nitrosopumilus maritimus*, oxidation

## Abstract

We investigate the potential for the globally distributed marine ammonia oxidising archaeon (AOA) *Nitrosopumilus maritimus* to oxidise iodide (I^−^), with the aim of identifying a key driver of seawater iodate (IO_3_^−^) renewal. Batch cultures of *N. maritimus* grew well in concentrations of 0.1 to 1 mM NH_4_^+^ and from 0.0001 to 1 mM I^−^. There was near 100% conversion of ammonium to nitrite over an 8‐day growth period. No loss of I^−^ or production of IO_3_^−^ was detected in cultures where I^−^ was added, indicating that *N. maritimus* is unable to drive I^−^ oxidation under the tested conditions. This contrasts with previous observations of I^−^ oxidation by ammonium oxidising bacteria (AOB). We explore whether differences between the metabolism of AOA and AOB could explain their differing actions on I^−^. *N. maritimus* cultures grown with the equivalent IO_3_^−^ concentrations also showed no reduction in [IO_3_^−^]. In addition, the growth of the *N. maritimus* culture was unaffected by inorganic iodine concentrations over 1000 times higher than in ambient seawater, suggesting a resilience to high iodine. These results suggest that AOA might have very little role in inorganic iodine turnover in the global ocean.

## Graphical abstract

This is the first ever study on the activity of the ammonia oxidising archaeon (AOA) *Nitrosopumilus maritimus* on inorganic iodine reactions. *N. maritimus* growth was clearly determined by increasing nitrite, but iodide and iodate concentrations remained constant throughout the experiment.

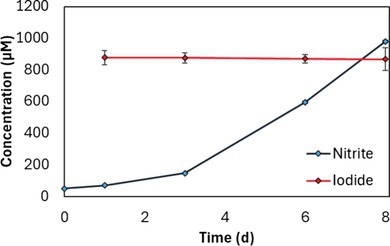

## 1 Introduction

Iodine has an important role in removing ozone from the troposphere, where it is both hazardous to human health (Zhang et al. 2019) and a significant greenhouse gas (Liu et al. 1987). In addition, iodine participates in particle formation reactions which subsequently affect the radiative balance of the Earth (McFiggans et al. 2010; Allan et al. 2015). The most abundant source of atmospheric iodine is sea‐air transfer, with an estimated flux of 10^11^–10^12^ g yr.^−1^ (Carpenter et al. 2013, 2021; Saiz‐Lopez et al. 2015), where molecular iodine or iodine oxidation products (HOI) occur as a direct result of the reaction of iodide (I^−^) with gaseous ozone (O_3_) at the sea surface (Carpenter et al. 2021). As a result, processes which determine the concentration of I^−^ in the surface ocean will directly affect subsequent tropospheric ozone reactions.

Thermodynamically, in oxic surface waters, iodate (IO_3_^−^) is the favoured form of iodine (Luther 2023); however, previous studies have found that I^−^ contributes up to 50% of the sea surface iodine pool (Tsunogai and Sase 1969; Truesdale et al. 2000; Bluhm et al. 2011; Chance et al. 2014). Concentrations of total inorganic iodine (I^−^ and IO_3_^−^) are generally within the range of 400–500 nM across the global surface ocean (Chance et al. 2014). Availability of I^−^ in the surface ocean is dependent upon environmental factors including biologically mediated production and loss, physical mixing, and advection from deeper water (Truesdale et al. 2000; Bluhm et al. 2010; Chance et al. 2014; Hepach et al. 2020; Wadley et al. 2020; Carpenter et al. 2021). Reduction of IO_3_^−^ to I^−^ is associated with the growth and senescence of marine phytoplankton (Moisan et al. 1994; Wong et al. 2002; Chance et al. 2007; Bluhm et al. 2010; Hepach et al. 2020). Processes driving the oxidation of I^−^ back to IO_3_^−^ must also be present to maintain the mass balance of iodine species in the oceans, but are not well understood (Truesdale et al. 2001; Hughes et al. 2021). Iodide oxidation rates in the oceans are estimated as months to years (Chance et al. 2014; Hardisty et al. 2020). The chemical conversion of I^−^ to IO_3_^−^ in seawater is extremely slow due to the thermodynamically unfavourable initial step of converting I^−^ to I_2_ at the pH of seawater and very low concentrations of suitable oxidants (Wong 1991; Luther 2023). Therefore, additional processes must be invoked to account for the estimated oceanic rates, and these are assumed to be biologically mediated.

Truesdale et al. (2001) and Žic et al. (2013) proposed that iodide oxidation is linked to biologically mediated nitrification processes in marine systems, and therefore has the potential to occur throughout the oceanic water column (Yool et al. 2007). Additionally, a number of iodide‐oxidising bacteria (genus *Iodidimonas*, closely related to genera *Roseovarius* and *Rhodothalassium*) have been isolated from seawater (Gozlan and Margalith 1973; Fuse et al. 2003; Arakawa et al. 2012; Iino et al. 2021), soils (Amachi et al. 2003; Li et al. 2012), groundwaters (Zhao et al. 2013; Lee et al. 2020), and brines (Amachi et al. 2005; Arakawa et al. 2012; Wakai et al. 2014; Khaing et al. 2019; Iino et al. 2021). Currently, three potential routes for the bacterial oxidation of iodide have been identified (Jiang et al. 2024): direct oxidation by bacterial multicopper oxidases (Amachi et al. 2005; Suzuki et al. 2012; Amachi and Iino 2022), direct oxidation by ammonia‐oxidising bacteria (Truesdale et al. 2001; Žic et al. 2013; Hughes et al. 2021), and indirect abiotic oxidation by extracellular reactive molecules (Li et al. 2012). In the first of these routes, bacteria are oxidising I‐ into molecular iodine (I_2_) using an oxygen‐dependent extracellular enzyme (Amachi et al. 2005; Iino et al. 2021). Despite this advance, as IO_3_^−^ production was not also demonstrated, it cannot be confirmed that these bacteria are also responsible for IO_3_^−^ renewal. To address the second route, based on water column observations (Truesdale et al. 2001; Žic et al. 2013), oxidation of I^−^ to IO_3_^−^ was suggested to be associated with nitrification, the two‐stage process of ammonium (NH_4_^+^) conversion to nitrate (NO_3_^−^) via nitrite (NO_2_^−^). Hughes et al. (2021) investigated ammonia‐oxidising bacteria (AOB) and NO_2_^−^ oxidising bacteria (NOB) to test this hypothesis and identify the stage at which I^−^ might be oxidised. IO_3_^−^ production was demonstrated in two AOB cultures (*Nitrosomonas* sp. Nm51 (C‐15) and *Nitrosococcus oceani* Nc10 (C‐107, ATCC 19707)) grown with added I^−^, but no difference was identified in cultures of NOB ( *Nitrospira marina* Nb‐295, *Nitrospina gracilis* 3/211 and *Nitrospina mobilis* Nb‐231). This suggests that only the ammonia‐oxidising microorganisms in that study were capable of oxidising I^−^ to IO_3_^−^.

Given that AOB may be able to oxidise I^−^, the possibility that ammonia oxidising archaea (AOA) can undertake the same role is an important question. Ammonia oxidising microorganisms are ubiquitous in terrestrial, aquatic and engineered environments. Ammonia oxidising archaea (AOA) are highly abundant, accounting for up to 40% of all prokaryotes in oceans globally (Karner et al. 2001). Potential participation of AOA in biogeochemical cycles other than those of nitrogen and carbon would be important given the ubiquitous distribution of several AOA species in global sea surface waters (Karner et al. 2001; Alves et al. 2018). It should be noted, however, that the data available on the molecular role of microbes in iodine oxidation is limited (Truesdale et al. 2001; Žic et al. 2013). None of these processes assigned to bacteria have been identified in archaea; this study is the first to attempt to assign a role to archaea within the marine iodine cycle.

The marine AOA *Nitrosopumilus maritimus* SCM1 is a widely used model organism and was originally isolated from a tropical marine aquarium in Seattle, USA (Könneke et al. 2005). *N. maritimus* utilises ammonia, and to a smaller extent cyanate, as a source of energy and nitrogen (Könneke et al. 2005; Kitzinger et al. 2019). Representatives of the genus *Nitrosopumilus* are ubiquitous, commonly found in oxic waters but also in oxygen‐minimum zones (Alves et al. 2018; Sollai et al. 2019). As the first ever isolated marine AOA and a representative of a highly abundant, globally distributed genus, *N. maritimus* is well suited for exploring the role of AOA in the iodine cycle and the impact of iodine on nitrification in the ocean. This study investigated the potential role of *N. maritimus* in the oxidation of I^−^ in seawater. Cultures of *N. maritimus* were grown under different ratios of NH_4_^+^ and I^−^ to constrain the stoichiometric ratio by which the rates of ammonia and I^−^ oxidation are associated, and to inform extrapolation to natural conditions.

## 2 Method

### 2.1 Cultivation of *N. maritimus* Cultures

*N. maritimus* SCM1 cultures were grown in Synthetic Crenarchaeota Media (Könneke et al. 2005) in the dark at 28°C for 7 days prior to inoculation for the experiments, in approximately 500 mL volume in 1 L acid‐washed Duran bottles. Immediately prior to inoculation, cultures were combined into a 2 L acid‐washed pre‐sterilised Duran bottle to ensure a uniform inoculum. All handling of the cultures was carried out aseptically. Routine monitoring for contamination by bacteria was performed using R2A plates and microscopy.

### 2.2 Preparation of Nitrogen and Iodine Stock Solutions

Stock solutions of NH_4_Cl (1 M; Thermo Scientific, 99+%), NaNO_2_ (1 M; Acros Organics, 99%), KI (1 M; Thermo Scientific 99+%) and KIO_3_ (0.25 M; Thermo Scientific 99.4%) were prepared gravimetrically in acid‐washed volumetric flasks with Milli‐Q water, and filter sterilised through 0.2 μm pore size sterile in‐line syringe filters (Fisherbrand, UK) into sterile 50 mL centrifuge tubes, sealed with parafilm and stored at 4°C.

### 2.3 Experimental Setup

Twenty‐five litres of Synthetic Crenarchaeota Medium were prepared in a non‐sterile manner in 5 L batches, without the addition of any nutrient stocks. Solutions of major media constituents (sodium pyruvate, HEPES, and FeEDTA) were made fresh, while trace metals solutions used pre‐prepared laboratory stocks. Sixty 500 mL acid‐washed bottles (Duran) were autoclaved in advance, and 400 mL of the prepared media was filter sterilised into each bottle through SUPOR PES sterile 0.2 μm pore size 47 mm diameter filters and a pre‐sterilised Nalgene bottle‐top filter rig.

Aliquots of NH_4_^+^, NO_2_^−^, I^−^ and IO_3_^−^ stock solutions were added to each bottle during the filter sterilising process to give the nominal final concentrations in Table 1. The experiment was divided into different treatment groups, each consisting of three *N. maritimus* cultures (replicates A, B and C) and one abiotic control (D) to confirm that any changes in iodine speciation were not due to a chemical or physical process. Groups 1–9 covered different ranges of I^−^ and NH_4_^+^ concentrations (from 0.0001 to 1 mM and 0.1 to 1 mM, respectively; Table 1), such that a range of I^−^ to NH_4_^+^ ratios was obtained. A range of concentrations was used (i) to identify if any I^−^ oxidation is associated with different stages of the growth curve, and (ii) inform extrapolation of results to ambient ratios of iodide: ammonium encountered in the oceans. While the highest I^−^ concentrations were significantly higher (up to 10,000×) than ambient sea level concentrations, the I^−^: NH_4_^+^ ratio did not exceed 1 in any condition. It should be noted that NH_3_^+^ concentrations were also at least 1000× higher than measured in open water (Bey et al. 2011), but are standard for the growth of *N. maritimus* in culture. Group 10 was a 1 mM NH_4_^+^ treatment with no I^−^ addition to evaluate whether I^−^ had any effect on the growth of *N. maritimus*. Groups 11 and 12 had two different concentrations of IO_3_^−^ (0.0001 and 1 mM, respectively) and 1 mM NH_4_^+^ to confirm any IO_3_^−^ generated in Groups 1–9 was not reconverted to I^−^ through reactions with hydroxylamine produced during the NH_4_^+^ oxidation process. Groups 13, 14 and 15 each consisted of a single abiotic treatment bottle of 1 mM I^−^ and 0.1–1 mM NO_2_^−^, to check there is no abiotic reaction between the NO_2_^−^ produced and I^−^. Media not used in the preparation of the treatment bottles was filter sterilised and stored for later use as a matrix blank during iodine analysis.

**TABLE 1:** Matrix of the additions of I^−^, IO_3_^−^, NH_4_^+^ and NO_2_^−^ to the treatment flasks.

	NH_4_^+^ concentration (μM)				NO_2_^−^ concentration (μM)		
I^−^ concentration (μM)		1000	500	100	1000	500	100
	1000	**1** (1)	**2** (2)	**3** (10)	**13** (1)	**14** (2)	**15** (10)
	500		**4** (1)				
	100	**5** (0.1)		**6** (1)			
	10	**7** (0.01)					
	1	**8** (0.001)					
	0.1	**9** (0.0001)					
	0	**10** (∞)					
IO_3_^−^ concentration (μM)	1000	**11** (1)					
	1	**12** (0.001)					

*Note:* For ease of identification, each condition is assigned a group number, shown in bold. Molar Nitrogen: Iodine ratios for each condition are given in brackets. Each group comprises triplicate *N. maritimus* cultures and an abiotic media only control, except for groups 13, 14 and 15 which had only one abiotic flask to check for IO_*3*_^*−*^ loss during processing.

To ensure successful growth of the *N. maritimus* culture, especially given the potential shock of introduction to high I^−^ concentrations, a relatively large inoculum volume was used: 50 mL inoculum of the previously prepared *N. maritimus* SCM1 stock was introduced to each flask aseptically (inoculum volume of 11%). To ensure the abiotic controls had the same chemical composition as the biotic treatment flasks, these flasks had the same volume (i.e., 50 mL) of 0.2 μm filtered inoculum added to each bottle. No inoculum was added to the abiotic NO_2_^−^ controls (flasks 13, 14 and 15), as these were solely to identify any chemical reactions with iodine. Bottles were incubated in a culture cabinet (Innova 44, New Brunswick Scientific, USA) in the dark at 28°C, over an 8‐day growth period.

### 2.4 Experimental Sampling

Due to the high inoculum volume, NO_2_^−^ was expected to be detectable and identical throughout all sample bottles immediately following inoculation. To this end, on day 0, only five treatment bottles were randomly sampled for NO_2_^−^ determination, with the assumption that unsampled flasks would have identical concentrations. Cultures were sampled for iodine speciation and NO_2_^−^ analyses on days 1, 3, 6 and 8, with pH measured on 1, 6 and 8. During sampling, to limit potential contamination of the culture bottles, a single combined sample of 50 mL was removed using a sterile pipette, which was subsequently filter sterilised through a 0.2 μm pore size sterile 25 mm diameter nylon in‐line syringe filter and aliquoted for pH, NO_2_^−^ and iodine speciation.

### 2.5 pH Analysis

For pH analysis, 5 mL of sample was removed and placed into a glass universal vial and analysed using a pH probe (Mettler Toledo FiveEasy, Australia), calibrated using standards prepared with technical buffer solution sachets of pH 4.01, 7.01 and 10.01 (Hanna instruments, UK).

### 2.6 NO_2_^−^

NO_2_^−^ was determined in filtered samples spectrophotometrically, using the Griess reagent method (Griess 1879; Lehtovirta‐Morley et al. 2014). Briefly, samples (100 μL) were treated with 20 μL sulfanilamide (0.5 g in 100 mL of 2.4 M HCl) and 20 μL N‐1‐naphthylethylenediamine dihydrochloride (NED) (0.3 g in 100 mL of 0.12 M HCl) to form a coloured azo dye, and the absorbance at 540 nm was measured using a spectrophotometer (VersaMax Tunable Microplate Reader, Molecular Devices, USA). NaNO_2_ standards were prepared within the range 0.7–50 μM, using the same NaNO_2_ stock as used in the experimental setup, diluted in Milli‐Q ultrapure water. Where samples fell outside this calibration range, samples were diluted quantitatively with Milli‐Q.

### 2.7 Iodine Speciation

Pre‐filtered sub‐samples for determination of iodine species (I^−^ and IO_3_^−^) were stored in sterile 15 mL centrifuge tubes and initially frozen at −80°C before storage for 3 weeks at −20°C prior to analysis. Iodide was measured using a modified version of the method of Jones et al. (2023) with a Thermo Scientific Dionex Inuvion Ion Chromatograph fitted with a Dionex IonPac AS24 2 × 250 mm analytical column with 2 × 50 mm guard column, both held at 35°C. The isocratic mobile phase was 0.2 M sodium chloride (NaCl, Sigma Aldrich BioXtra ≥ 99.5%) with 100 mM orthophosphoric acid (H_3_PO_4_), at a flow rate of 0.45 mL min^−1^. Detection of the I^−^ ion used a UV Diode Array Detector (Agilent 1260 Infinity II, USA), measuring the absorbance at wavelength 226 nm (reference wavelength 360 nm) in a flow cell with 4 μL volume in a 60 mm path length (G4212‐60007). Samples were injected into the system by a Thermo Scientific Dionex AS‐AP autosampler. Calibration standards were prepared using the autosampler by serial dilution of a 0.1 M KI stock standard (Thermo Scientific 99+%) prepared in 0.4 M NaCl.

Given the wide concentration range of the experimental treatments, samples were analysed on two different calibration ranges: samples where I^−^ was ≤ 1 μM (groups 8, 9, 10, 11 and 12) were analysed directly on a calibration range 0.01–2 μM with the ion chromatograph equipped with a 50 μL sample loop. Higher concentration samples (groups 1–7 and 13–15) were analysed on a 100–10,000 μM I^−^ calibration with a 5 μL sample loop, with samples diluted with 0.4 M NaCl where appropriate to be within the calibration range.

Limits of blank (LOB), limits of detection (LOD) and limits of quantification (LOQ) were calculated for both low and high calibration ranges according to the definitions given in Armbruster and Pry (2008) and Boqué and Heyden (2009), respectively (Table 2). The chromatographic method was not affected by the high and concentrations of NO_2_^−^ present in the cultures, because there was good separation of the two ions: NO_2_^−^ elutes at 3.5 min, while I^−^ elutes at 9.5 min. Examination of I^−^ standards in the high calibration range (100–10,000 μM) spiked with identical concentrations of NO_2_^−^ showed NO_2_^−^ had no effect on the I^−^ signal at 226 nm. A number of samples (*n* = 7) were analysed in triplicate, with the mean and standard deviation calculated. The mean percent standard deviation from these samples was 2.9%.

**TABLE 2:** Limits of blank (LOB), limit of detection (LOD) and limit of quantification (LOQ) for the I^−^ analysis by ion chromatography, for both low and high calibrations.

	Equation	Iodide low calibration	TII low calibration	Iodide high calibration	TII high calibration	References
	Calibration Range (nM)	10–1250	10–1250	100–10,000	100–10,000	
LOB (nM)	LOB = Mean_blank_ ± 1.645 (SD_blank_)	0.09	0.12	0.023	N/A	(Armbruster and Pry 2008)
LOD (nM)	3 × (SD_LowestConcentratIonStandard_)	5.0	20.3	70.0	43.2	(Boqué and Heyden 2009)
LOQ (nM)	10 × (Mean_LowestConcetrat_ _io_ _nStandard_)	15.0	61.2	212.3	130.9	(Boqué and Heyden 2009)

Total inorganic iodine (TII; consisting of I^−^ and IO_3_^−^) was determined as I^−^ following treatment with hydroxylamine (NH_2_OH–HCl; Sigma‐Aldrich Reagent Plus, 99%; final sample concentration of 35 mM; Jones et al. 2023) to reduce all oxidised inorganic iodine forms (predominantly IO_3_^−^) to I^−^. TII samples were calibrated using a 0.1 M potassium IO_3_^−^ stock standard (KIO_3_, Thermo Scientific 99.4%), with the calibration range identical to that used for the I^−^. Serial dilutions and addition of the reagent to standard was carried out using the autosampler, while reagent was added manually to samples. Calibrations of I^−^ and TII were compared and found to have identical slope, indicating ~100% recovery of IO_3_^−^ as I^−^. Calculated LOD and LOQ for TII were higher than those for I^−^ in the lower calibration range, due to a higher SD (Table 2). IO_3_^−^ in the samples was calculated by subtracting the I^−^ concentrations from the TII, and the propagated error was calculated from the standard deviation of the I^−^ and TII replicate samples. IO_3_^−^ standards spiked with identical concentrations of NO_2_^−^ on the high calibration range (100–10,000 μM) showed NO_2_^−^ had zero effect on the quantitative reduction of IO_3_^−^ to I^−^.

## 3 Results

### 3.1 Microbial Growth and Accumulation of NO_2_ ^−^

**FIGURE 1:**
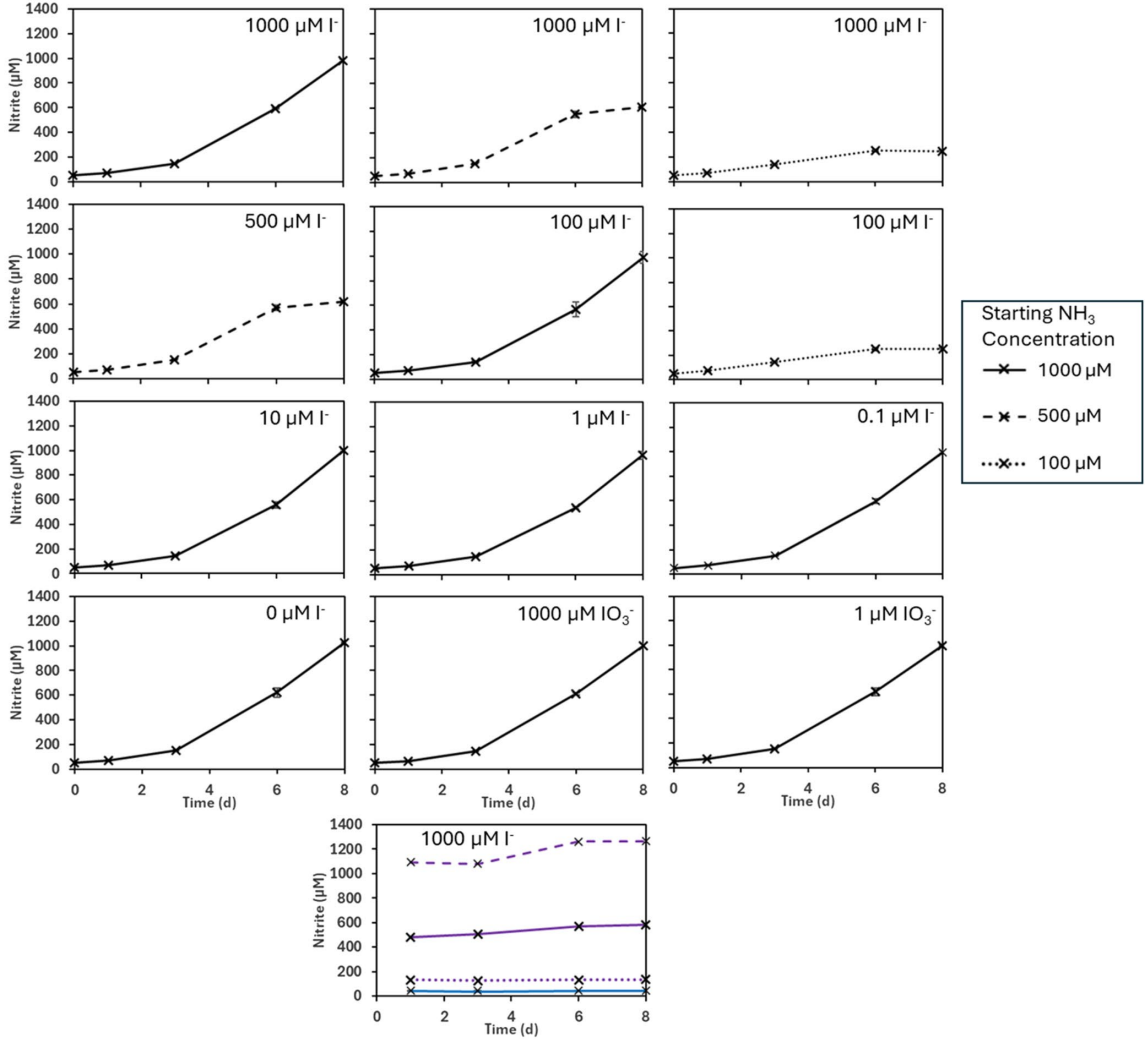
NO_2_^−^ measured in all *N. maritimus* cultures over the 8‐day incubation. Panel numbers correspond to the groups given in Table 1, and I^−^/IO_3_^−^ concentrations are shown. The legend shows the starting NH_4_^+^ concentration: Solid black lines correspond to cultures with 1 mM NH_4_^+^ addition (Groups 1, 5, 7, 8, 9, 10, 11, 12), dashed black lines correspond to cultures with 0.5 mM NH_4_^+^ addition (Groups 2, 4) and dotted black lines correspond to cultures with 0.1 mM NH_4_^+^ addition (Groups 3, 6). All panels 1–12 show the average, with error bars showing the standard deviation of triplicate cultures. Panel 13 shows the abiotic controls for 1 mM (purple dashed), 0.5 mM (purple solid) and 0.1 mM (purple dotted) NO_2_^−^, as well as the mean of all the NH_4_^+^ abiotic controls of all treatment groups (blue solid).

Nitrite accumulation was used as a proxy for growth of ammonia oxidising archaea, as nitrite is the end‐product of ammonia oxidation and has been shown to increase proportionally with cell number (Könneke et al. 2005; Lehtovirta‐Morley, Ross, et al. 2016). No cell count data are available from this study. The mean starting concentration at day 0 in the *N. maritimus* cultures was 52 (±0.3) μM NO_2_^−^ (Figure 1, all panels). No NO_2_^−^ was added to the media (except for groups 13–15, Table 1), and the NO_2_^−^ measured at day 0 originated from the culture inoculum, which was in late exponential growth phase. It is assumed that due to the late growth stage of the inoculum, ammonia concentrations would be minimal. Mean NO_2_^−^ concentrations in the abiotic controls of treatments containing NH_4_^+^ (Groups 1–12, Table 1) remained stable throughout the growth period and were measured at 46 (±1) μM on day 1 through to 44 (±3) μM on day 8 (Figure 1). Mean concentrations in the NO_2_^−^ abiotic controls across the 8‐day period were also relatively stable (1175 ± 102, 535 ± 48 and 132 ± 5, respectively), but did show a slight increase between days 3 and 6 (Figure 1, panel 13).

All *N. maritimus* cultures converted NH_4_^+^ to NO_2_^−^, with growth rates (0.35 ± 0.01 d^−1^) equivalent in all cultures up until when ammonia was depleted. Those cultures with only 100 μM NH_4_^+^ entered stationary phase following day 3 (final day NO_2_^−^ 252 ± 6 μM; Figure 1, panels 3 and 6), those with 500 μM after day 6 (final day NO_2_^−^ 613 ± 11 μM; Figure 1, panels 2 and 4), and the highest concentration cultures at 1 mM NH_4_^+^ around day 8 (995 ± 24 μM; Figure 1). There was no difference in NO_2_^−^ concentrations between replicate cultures, or between groups with the same starting NH_4_^+^ concentration but different concentrations of iodine anions. Of particular note, no difference in NO_2_^−^ concentration was observed between those treatments with I^−^ concentrations ranging from zero to 1 mM (final day 8 NO_2_^−^ concentration range 970–1025 μM; Figure 1, panel 1 through panel 10). Similarly, there was no effect from increasing IO_3_^−^ concentration on *N. maritimus* growth, where the day 8 NO_2_^−^ concentrations in groups 11 (1 mM IO_3_^−^) and 12 (1 μM IO_3_^−^) were 998–1002 μM (Figure 1, panels 11 and 12).

### 3.2 pH in the Cultures

**FIGURE 2:**
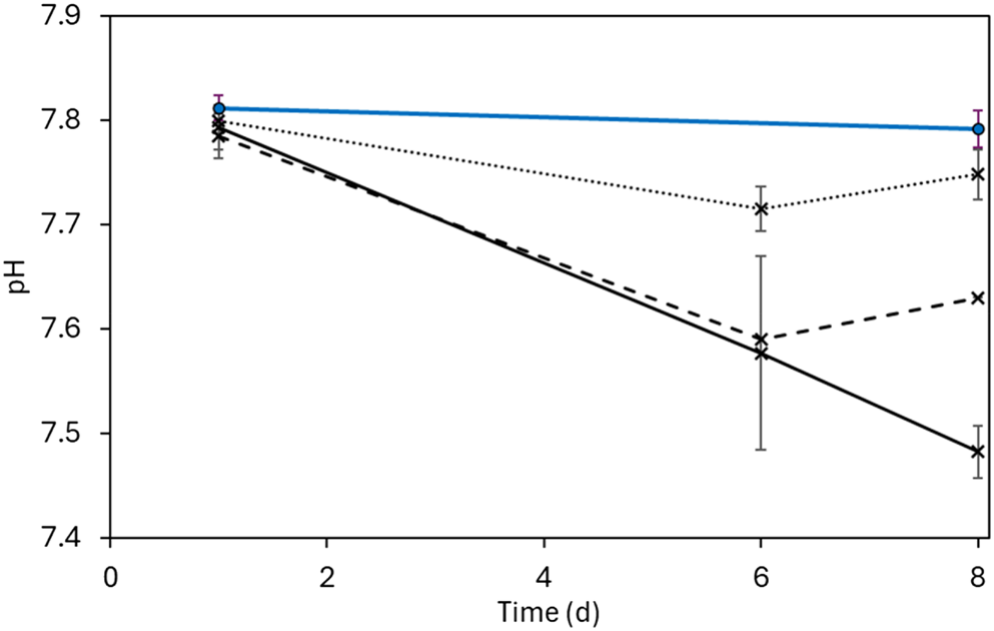
pH in *N. maritimus* cultures during the experiment, showing the mean of cultures with 1 mM NH_4_^+^ addition (solid black), mean of cultures with 0.5 mM NH_4_^+^ addition (dashed black) and mean of cultures with 0.1 mM NH_4_^+^ addition (dotted black), compared to the average pH in the abiotic controls (solid blue). Error bars represent standard deviation for all relevant cultures.

pH in the cultures decreased over the 8 days of the experiment in all biotic treatments, but no treatment had declined below 7.41 by day 8. Increasing cell density of *N. maritimus* resulted in lower pH at day 8, and the mean pH in cultures grown with 100 μM NH_4_^+^ was 7.75 (±0.02), mean with 500 μM NH_4_^+^ was 7.63 (±0.0) and mean with 1000 μM NH_4_^+^ was 7.48 (±0.02) on day 8 (Figure 2). At no time did the culture pH turn acidic, which is important as NO_2_^−^ is known to react with I^−^ at pH < 5.5 (O'Driscoll et al. 2008). The abiotic cultures showed little change, with the mean of all flasks at 7.80 (±0.02).

### 3.3 Iodide Concentrations and Monitoring of Iodide Consumption

**FIGURE 3:**
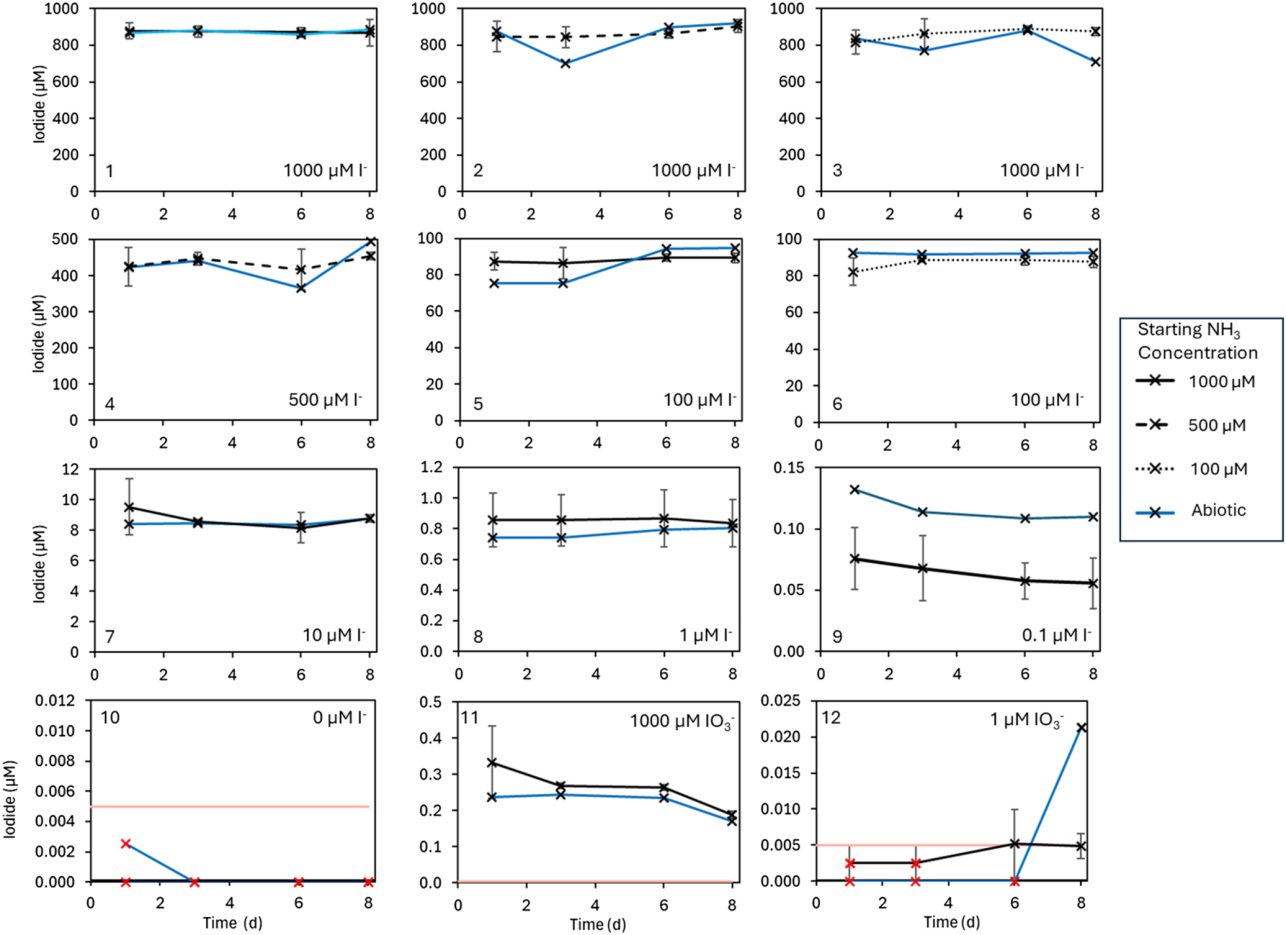
I^−^ measured in all *N. maritimus* cultures over the 8‐day incubation. Panel numbers correspond to the groups given in Table 1, and I^*−*^ */*IO_3_^−^ concentrations are shown. The legend shows the starting NH_4_^+^ concentration: Solid black lines correspond to cultures with 1 mM NH_4_^+^ addition (Groups 1, 5, 7, 8, 9, 10, 11, 12), dashed black lines correspond to cultures with 0.5 mM NH_4_^+^ addition (Groups 2, 4) and dotted black lines correspond to cultures with 0.1 mM NH_4_^+^ addition (Groups 3, 6). Average of triplicate cultures is shown with error bars representing the standard deviation. Solid blue lines represent the abiotic controls within each treatment group, and data points highlighted in red were those which were below the calculated detection limit (given by the pale red line).

There was no change in the I^−^ concentration in any of the *N. maritimus* cultures over the 8 days of the incubation (Figure 3, panel 1 through panel 9). In addition, for each I^−^ concentration used, no differences were observed between different NH_4_^+^ levels. Measured I^−^ concentrations were slightly lower than the nominal target concentrations (Table 1) due to the dilution effect of the culture inoculum addition, which did not contain I^−^ (Table S1) The concentrations of I^−^ measured in the abiotic controls were within the standard deviation of the cultures. Concentrations in the control with no I^−^ addition (Group 10, Table 1) were below the detection limit (5 nM; Table 2), which indicated that any I^−^ contamination in the inorganic salts (predominantly NaCl) used for the media was minimal. *N. maritimus* is not routinely cultured in media containing iodine and there is therefore no regular exposure of these organisms to I^−^ during the standard handling in the laboratory. A small amount of I^−^ was identified in the flasks amended with IO_3_^−^(groups 11 and 12; Figure 3, panels 11 and 12): 0.17–0.45 μM at 1000 μM IO_3_^−^ and 0.0007–0.011 μM at 1 μM IO_3_^−^. These represent below 1% of the added IO_3_^−^ and are considered a minor contaminant from the IO_3_^−^ addition due to the presence from day 1 and similar values in the abiotic flasks.

### 3.4 Monitoring of Total Inorganic Iodine and Iodate Concentration

**FIGURE 4:**
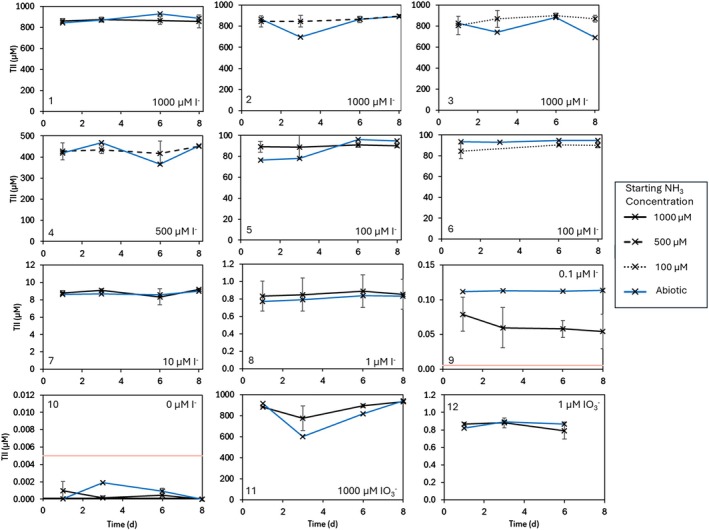
Total Inorganic Iodine (TII) measured in all *N. maritimus* cultures over the 8‐day incubation. Panel numbers correspond to the groups given in Table 1, and I^−^/IO_3_^−^ concentrations are shown. The legend shows the starting NH_4_^+^ concentration: Solid black lines correspond to cultures with 1 mM NH_4_^+^ addition (Groups 1, 5, 7, 8, 9, 10, 11, 12), dashed black lines correspond to cultures with 0.5 mM NH_4_^+^ addition (Groups 2, 4) and dotted black lines correspond to cultures with 0.1 mM NH_4_^+^ addition (Groups 3, 6). Average of triplicate cultures is shown with error bars representing the standard deviation. Solid blue lines represent the abiotic controls within each treatment group, and data points highlighted in red were those which were below the calculated detection limit (given by the pale red line).

Total inorganic iodine (I^−^ + IO_3_^−^) did not show any changes over the course of the experiment (Figure 4, panel 1 through panel 12). Across groups 1–9, I^−^ comprised 99.9% of the TII signal across the 8‐day incubation period. Similarly, in those groups with iodate added (groups 11 and 12; Figure 4, panels 11 and 12), 99.9% of the TII signal was a result of IO_3_^−^, which did not change across the week of the incubation.

Iodate concentrations are calculated as the difference between TII and iodide concentrations. Calculated IO_3_^−^ concentrations were too small to be quantifiable in this study: calculated IO_3_^−^ was either below the detection limit of the ion chromatograph for TII and I^−^ measurements, or the error in IO_3_^−^ concentration (calculated by propagation of the errors in the I^−^ and TII measurements) was much higher than the calculated concentrations. A paired t‐test to compare the concentrations of I^−^ and TII in I^−^ added groups (Groups 1–9) showed no significant difference between the analyses (df = 157, *p* < 0.01). Given this, the calculated iodate concentrations are not shown.

## 4 Discussion

### 4.1 Tolerance of Extreme Iodide and Iodate Concentrations by *N. maritimus*

Increasing I^−^ concentrations to up to 1 mM had no effect on the growth of *N. maritimus* and the growth rates between day 0 and day 3 were identical in all treatments (0.35 ± 0.01 d^−1^). The length of the exponential growth period was only dependent upon the starting NH_4_^+^ concentration and there was no impact from increasing I^−^ or IO_3_^−^ concentrations. Indeed, with the treatments of 1 mM, these concentrations were more than 10,000 times higher than the median surface seawater I^−^ concentration (77 nM; Chance et al. 2014) that *N. maritimus* would be exposed to in the ocean. Across the vast majority of the oxygenated oceans, total dissolved iodine is around 450 nM, with less than half as I^−^ (Chance et al. 2014) and the majority as IO_3_^−^. Rarely, much higher I^−^ concentrations (around 1000 nM) are observed in seawater. These features appear to be associated with plumes from sediments in low oxygen environments (Farrenkopf and Luther III 2002; Cutter et al. 2018; Chance et al. 2020).

Archaea belonging to the family *Nitrosopumilales* are ubiquitous across the world's oceans (Alves et al. 2018) and the ability of *N. maritimus* to withstand I^−^ concentrations of 1 mM, 10,000 times higher than the ambient seawater concentration, is surprising given that marine AOA would rarely be exposed to such conditions. If other archaea are similarly tolerant of I^−^, it may allow for colonisation of high I^−^ niches such as iodine‐rich brines and groundwaters, which have I^−^ concentrations around 1 mM (Amachi et al. 2005). Indeed, when Archaea were first identified, they appeared at first to be a narrowly distributed group isolated in extreme environments, including extreme halophiles, extreme thermophiles, and thermoacidophiles (DeLong 2021). Included in these diverse species are methanogens isolated from hypersaline ponds and iodine‐rich aquifers (Urai et al. 2021) where iodine concentrations were 1.2 mM. Interestingly, several species of I^−^ oxidising bacteria have also been isolated from iodide‐rich natural gas brines (Amachi et al. 2005). However, there is currently no evidence associating these extremophile Archaea with the open‐ocean dwelling *N. maritimus*. Factors which determine the environmental distribution of archaea include salinity (Blainey et al. 2011; Lehtovirta‐Morley 2018). The salinity range of *N. maritimus* SCM1 is relatively limited and reflects its seawater habitat (optimum 32‰–37‰, range 16‰–55‰; Bayer, Pelikan, et al. 2019). Therefore, iodine tolerance appears to be a feature of some archaea which is unrelated to overall high salinity tolerance.

### 4.2 Absence of Iodide Oxidation in *N. maritimus* Cultures

The absence of a detectable decrease in iodide or increase in iodate in any of the biotic treatments suggests that the AOA *N. maritimus* is not a significant driver of I^−^ oxidation in the oceans. In the majority of treatments, the cultures were in exponential growth phase throughout, while the treatment groups with only 100 μM addition of NH_4_^+^ were in stationary phase by the conclusion of this experiment. Therefore, iodide oxidation was not associated with different growth phases of *N. maritimus*.

No differences were observed between I^−^ treatments, indicating that an elevated I^−^: NH_4_^+^ ratio did not enhance conversion of I^−^ to IO_3_^−^, compared to a 1:1 or reduced I^−^: NH_4_^+^ ratio. Ratios in the experiment were influenced by natural stoichiometric ratios of I^−^: NH_4_^+^, as found in surface ocean environments. Seawater surface ammonium concentrations mid Atlantic near 0° Latitude were below 10 nM (Johnson et al. 2008), corresponding to I^−^concentrations over 100 nM (Chance et al. 2014), giving I^−^: NH_4_^+^ of 10:1 or higher. In the North Atlantic, the I^−^: NH_4_^+^ is approximately 1:1 with NH_4_^+^ and I‐ concentrations both near 50 nM; (Rees et al. 2006; Chance et al. 2014), whereas Southern Ocean NH_4_^+^ concentrations of over 500 nM (Smith et al. 2022) correspond to much lower I^−^ of 10–50 nM (Chance et al. 2014) giving ratios of 1:10 or higher. In coastal regions such as the North Sea, ammonium concentrations measured over 2500 nM (Sørensen et al. 2003), alongside I^−^ of 50–100 nM (Chance et al. 2014), giving I^−^: NH_4_^+^ ratios of 1:50 or higher. Replication of these ratios during this study showed I^−^ oxidation was not closely associated with the reactions and uptake of NH_4_^+^ by the AOA. In addition, no evidence was identified that I^−^ was utilised in the absence of NH_4_^+^ during stationary phase cultures. In contrast to this study, Hughes et al. (2021) found IO_3_^−^ production by cultures of AOB grown under high I^−^. It is possible that small changes in iodine speciation (a few tens of nanomolar) potentially occurred within our cultures but were not detectable as they were less than the analytical error.

The controls included in the experimental design allowed us to confirm that the absence of changes in iodine speciation is not the result of additional confounding processes. NH_2_OH is produced by *N. maritimus* as a step in the NH_4_^+^ oxidation process (Vajrala et al. 2012) and is also known to reduce oxidised inorganic iodine species (Jones et al. 2023). There was therefore concern that if *N. maritimus* was responsible for the oxidation of I^−^ to IO_3_^−^, then generation of NH_2_OH in the cultures could immediately reduce any de novo IO_3_^−^ back to I^−^, leading to no detectable change in concentrations. Concentrations of NH_2_OH in *N. maritimus* cultures are expected to be low (Vajrala et al. 2012) with the assumption it is either tightly enzyme‐bound or rapidly converted to other metabolites. Liu et al. (2017) found no evidence of extracellular NH_2_OH from three other species of AOA, and it is possible that *N. maritimus* acts similarly. However, to confirm this here, cultures were prepared (groups 11 and 12), with IO_3_^−^ concentrations equivalent to those hypothetically produced during the *N. maritimus* growth assuming a 100% oxidation of I^−^ across the growth period. I^−^ was not observed to increase in these treatments (Figure 3), and IO_3_^−^ concentrations did not show a significant decrease (as calculated from I^−^ subtracted from TII; Figure 4). IO_3_^−^ was responsible for 100% of the total inorganic iodine throughout all sampling days in these treatments. This suggests IO_3_^−^ was not being reduced by NH_2_OH in the AOA cultures and the absence of I^−^ loss and IO_3_^−^ formation in the I^−^ treatments is unlikely to be explained by the reaction of IO_3_^−^ with NH_2_OH produced during the NH_4_^+^ oxidation process. The absence of IO_3_^−^ loss from those flasks with IO_3_^−^ added instead of I^−^ (groups 11 and 12) also implies that the reduction of IO_3_^−^ to I^−^ in the surface ocean is also unlikely to be caused by AOA. In contrast, AOB have been found to release NH_2_OH into the surrounding media, and it is possible this may have counteracted IO_3_^−^ production in the AOB experiments reported by Hughes et al. (2021).

### 4.3 The Role of Microbial Processes in Iodide Oxidation

To consider why AOA might differ to AOB in their ability to oxidise I^−^, we examine here the various routes to iodide oxidation that have been identified in bacteria, and compare them to known features of AOA metabolism.

#### 4.3.1 Potential Interactions Between Iodide and the Ammonia Oxidation Pathway

Hughes et al. (2021) showed that cultures of the marine ammonia‐oxidising bacteria *Nitrosomonas* sp. (Nm51) and *Nitrosococcus oceani* (Nc10) oxidised I^−^ to IO_3_^−^. Their results appeared to confirm the hypothesis developed from field observations (Truesdale et al. 2001; Žic et al. 2013) that iodide oxidation is linked to nitrification, and suggested this is due to the activity of ammonia oxidising bacteria but did not identify the mechanism. During ammonia oxidation, ammonia is first oxidised to hydroxylamine by the membrane‐bound ammonia monooxygenase (AMO) enzyme (Lehtovirta‐Morley 2018). AMO is found in all known autotrophic ammonia oxidisers, including archaea, and the bacterial AMO has a wide substrate range. The bacterial AMO can oxidise a range of substrates, including alkanes, alkenes, halogenated hydrocarbons, sulphides and aromatic compounds (Hyman et al. 1990; Rasche et al. 1991; Juliette et al. 1993; Keener and Arp 1993, 1994). Hughes et al. (2021) suggested that AMO might act on I^−^ as an alternate to ammonia as a first step towards IO_3_^−^ formation. Two reasons have been postulated for this action: firstly, the outer membrane position of AMO allows for reactions with I^−^ without the requirement of active uptake (Hughes et al. 2021); secondly, Luther (2023) suggested the 2‐electron process occurring during the ammonia to NH_2_OH can mirror the 2‐electron transfer required to convert I^−^ to HOI. It is apparent from the results here that I^−^ cannot be considered a substrate for *N. maritimus* AMO, implying either that bacterial and archaeal AMO enzymes differ in their ability to oxidise iodide, or that AMO more generally is not involved in iodide oxidation. It is known that the archaeal AMO is divergent from that of the AOB (Lehtovirta‐Morley 2018), with lower apparent substrate affinity (*K*_m(app)_) for NH_4_^+^ than AMO from bacterial strains (Jung et al. 2022). There are currently no known alternative substrates of archaeal AMO, which is consistent with significant functional difference between archaeal and bacterial AMO, but may also reflect that, as a more recently discovered group, there is a need for further research on AOA functions. Alternatively, the AMO may not be involved in I^−^ oxidation. Known substrates for the AMO, and other related CuMMO superfamily enzymes, include only uncharged substrates, and it is therefore unclear whether the negatively charged I^−^ could be oxidised by the AMO, even overcoming the significant size difference. The downstream pathway of the ammonia oxidation differs between AOA and AOB (Lehtovirta‐Morley 2018), but the enzymes participating in it are not well understood. In AOB, hydroxylamine is oxidised in the periplasm by hydroxylamine oxidoreductase (Arp et al. 2002). While hydroxylamine has been reported as the intermediate in *N. maritimus* (Vajrala et al. 2012) the downstream pathway from AMO in AOA remains unknown (Walker et al. 2010; Lehtovirta‐Morley, Sayavedra‐Soto, et al. 2016). It is not yet understood whether either of these pathways could act on iodine species to complete the oxidation of iodide to iodate (Liu et al. 2017). As understanding of these processes improves, it is worth revisiting the question of I^−^ oxidation reactions, particularly those in the AOB.

#### 4.3.2 Oxidation by Multicopper Oxidases

Iodide oxidation pathways independent of nitrogen metabolism have also been identified in marine bacteria. Two strains of I^−^ oxidising bacteria, *Iodidimonas* sp. Q‐1 and *Roseovarius* sp. A‐2, use multicopper iodide oxidases, comprised of at least two subunits, IoxA and IoxC, to oxidise I^−^ extracellularly (Amachi et al. 2005; Suzuki et al. 2012; Shiroyama et al. 2015; Amachi and Iino 2022). Following the initial I^−^ oxidation to HOI, the subsequent reaction to IO_3_^−^ can occur spontaneously (Amachi and Iino 2022; Luther 2023; Jiang et al. 2024). Homologues of *ioxA* and *ioxC*, genes encoded for the subunits of the multicopper iodide oxidase, were identified in other I^−^ oxidising bacteria (Suzuki et al. 2012; Shiroyama et al. 2015; Lee et al. 2020) and are also present in some species of *Nitrosomonas* AOB, although it is not known whether the entire operon is present in these species of AOB. The function of these homologues in AOB has not been verified but should be explored by future research; where present, they might permit simultaneous iodide and ammonium oxidation via separate mechanisms but a single organism. The presence of these homologues has not been investigated in *N. maritimus*, but the absence of I^−^ oxidation suggests this is not a method of oxidation these species undertake.

#### 4.3.3 Extracellular Abiotic Processes

Accumulation of extracellular reactive oxygen species such as superoxide and H_2_O_2_ occurs in AOB cultures (Diaz et al. 2013; Hansel and Diaz 2020), whereas even nanomolar concentrations of H_2_O_2_ can inhibit growth of AOA (Kim et al. 2016; Tolar et al. 2016; Bayer, Vojvoda, et al. 2019). To limit this effect, pyruvate is often added to AOA cultures, including those described in this work, to scavenge it (Qin et al. 2014). Studies on bacterial isolates showed the oxidation of I^−^ to tri‐iodide (I_3_^−^) in the presence of H_2_O_2_, facilitated by organic acids (Li et al. 2012). The absence of H_2_O_2_ in the *N. maritimus* cultures (Martens‐Habbena et al. 2009; Qin et al. 2014; Kim et al. 2016) will prevent this indirect I^−^ oxidation, but may explain the oxidation in the AOB cultures of Hughes et al. (2021).

### 4.4 Future Work

It is clear that many unanswered questions remain about the potential role of ammonium oxidizers in the iodine cycle, and further studies of both AOA and AOB are required. We propose a repeat of the studies with *Nitrosomonas* and *Nitrosococcus oceani* , alongside other species of AOA and/or AOB, including strains containing the homologues of the *iox* operon multicopper oxidases (IoxA and IoxC) (e.g., *Iodidimonas nitroreducens*). It is possible that the apparent difference in the ability of AOA (this study) and AOB (Hughes et al. 2021) to oxidise I^−^ arises from differences in the physiological state of the cultures used, as growth in the AOA cultures was normal, while the AOB studied by Hughes et al. (2021) exhibited only very modest increases in cell density and nitrite concentration, suggesting they were not growing as would typically be expected. Therefore, future experiments should ensure optimal growth in all cultures. Addition of pyruvate to bacterial cultures could also be made to ascertain whether extracellular reactions with H_2_O_2_ are important. Although Hughes et al. (2021) conducted tests to confirm the accuracy of the spectrophotometric iodate method at the nitrite concentrations they encountered, high levels of NO_2_^−^ production are likely to interfere with the spectrophotometric determination of iodate (Chapman and Liss 1977), and therefore use of alternative analytical methods such as ion chromatography is recommended.

## 5 Conclusions

This study has shown that the ammonia oxidising archaeon *N. maritimus* does not significantly contribute to the oxidation of I^−^ to IO_3_^−^ in cultures. *N. maritimus* is considered a model marine AOA species, but there is also potential for strain‐ and species‐specific differences between *N. maritimus* cultures and other surface seawater dwelling AOA, so it cannot be stated with certainty that AOA as a whole are not contributing to I^−^ oxidation. The findings of this study contrast with previous work which suggested AOB might contribute significantly to marine iodide oxidation (Hughes et al. 2021). Understanding the reasons for this difference is hindered by a lack of understanding of both the mechanism by which AOB may oxidise iodide and the limited knowledge of how metabolism differs in AOA and AOB. Further research into potential interactions between iodine and nitrogen metabolism in marine microbes is required. It is suggested that this study should be repeated with alternative species of AOA, AOB and NOB. The lack of I^−^ and IO_3_^−^ toxicity in *N. maritimus* is surprising, even at concentrations over 10,000× higher than those found in surface seawater. This is consistent with the ubiquity of *N. maritimus* in the oceans and indicates that *N. maritimus* will not be affected by fluctuations in iodine speciation or changes in I^−^ in future ocean scenarios.

## Author Contributions

A.L.W., R.C. and L.E.L.M. planned the experimental design. A.L.W. and B.O.R. undertook the experimental processes and analyses. M.W. and A.W. set up and developed the iodine analytical procedures for the ion chromatograph system. A.L.W., R.C. and B.O.R. prepared the manuscript, with editing and comments from L.E.L.M. and L.J.C. L.J.C. has overall project responsibility.

## Conflicts of Interest

The authors declare no conflicts of interest.

## Supporting information


**Table S1:** Measured concentrations of NO_2_^−^ in cultures of *N. maritimus* SCM1 grown under a range of iodine and nitrogen ion concentrations; experiment number refers to conditions listed in Table 1. Letters A–C in the experiment number refer to the biotic treatments, D refers to the abiotic control flasks.
**Table S2:** Target and actual measured concentrations of I^−^ in cultures of *N. maritimus* SCM1 grown under a range of iodine and nitrogen ion concentrations; experiment number refers to conditions listed in Table 1. Letters A–C in the experiment number refer to the biotic treatments, D refers to the abiotic control flasks.
**Table S3:** Target and actual measured concentrations of TII added to the biotic cultures during the Archaea growth experiment, alongside the actual measurements in the cultures following addition of the inoculum.

## Data Availability

The data that supports the findings of this study are available in the Supporting Information of this article.
