# Supplementary material for: The Ammonia Oxidising Archaeon Nitrosopumilus maritimus Does Not Alter Iodine Oxidation State in Oxic Seawater
Source: Environ Microbiol Rep. 2025 Aug 10;17(4):e70168. doi: 10.1111/1758-2229.70168 (PMC12335899; doi:10.1111/1758-2229.70168)
Supplement: Supplementary file 1 — Table S1: Measured concentrations of NO2 − in cultures of N. maritimus SCM1 grown under a range of iodine and nitrogen ion concentrations; experiment number refers to conditions listed in Table 1. Letters A–C in the experiment number refer to the biotic treatments, D refers to the abiotic control flasks. Table S2: Target and actual measured concentrations of I− in cultures of N. maritimus SCM1 grown under a range of iodine and nitrogen ion concentrations; experiment number refers to conditions listed in Table 1. Letters A–C in the experiment number refer to the biotic treatments, D refers to the abiotic control flasks. Table S3: Target and actual measured concentrations of TII added to the biotic cultures during the Archaea growth experiment, alongside the actual measurements in the cultures following addition of the inoculum. [file EMI4-17-e70168-s001.docx]

## Supplementary

Supplementary Table 1. Measured concentrations of NO_2_^-^ in cultures of N. maritimus SCM1 grown under a range of iodine and nitrogen ion concentrations; experiment number refers to conditions listed in table 1. Letters A-C in the experiment number refer to the biotic treatments, D refers to the abiotic control flasks.

| Experiment No | Starting NH_4_^+^ concentration (µM) | Measured Concentration (µM)  day 0 Mean (±Stdev) | Measured Concentration (µM)  day 1 Mean (±Stdev) | Measured Concentration (µM)  day 3 Mean (±Stdev) | Measured Concentration (µM)  day 6 Mean (±Stdev) | Measured Concentration (µM)  day 8 Mean (±Stdev) |
| --- | --- | --- | --- | --- | --- | --- |
| 1 A-C | 1000 | 51.7 (±0.3) | 71.5 (±1.4) | 147.1 (±4.1) | 596.0 (±7.2) | 980.5 (±8.4) |
| 1 D | 1000 | 51.7 (±0.3) | 46 | 43 | 43 | 43 |
| 2 A-C | 500 | 51.7 (±0.3) | 69.9 (±5.3) | 151.7 (±2.2) | 547.9 (±26.0) | 605.4 (±5.9) |
| 2 D | 500 | 51.7 (±0.3) | 47 | 43 | 43 | 42 |
| 3 A-C | 100 | 51.7 (±0.3) | 71.7 (±1.1) | 138.2 (±3.3) | 254.3 (±10.4) | 248.7 (±5.2) |
| 3 D | 100 | 51.7 (±0.3) | 46 | 43 | 43 | 44 |
| 4 A-C | 500 | 51.7 (±0.3) | 74.9 (±1.1) | 155.4 (±1.9) | 570.7 (±20.8) | 619.8 (±9.3) |
| 4 D | 500 | 51.7 (±0.3) | 47 | 43 | 43 | 44 |
| 5 A-C | 100 | 51.7 (±0.3) | 72.4 (±1.4) | 141.9 (±9.0) | 566.4 (±57.4) | 987.5 (±48.5) |
| 5 D | 100 | 51.7 (±0.3) | 47 | 45 | 44 | 45 |
| 6 A-C | 1000 | 51.7 (±0.3) | 72.4 (±0.1) | 146.2 (±0.8) | 251.0 (±12.0) | 254.4 (±6.5) |
| 6 D | 1000 | 51.7 (±0.3) | 46 | 44 | 44 | 44 |
| 7 A-C | 1000 | 51.7 (±0.3) | 71.2 (±1.2) | 147.6 (±3.9) | 559.0 (±24.6) | 1001.3 (±9.5) |
| 7 D | 1000 | 51.7 (±0.3) | 45 | 42 | 43 | 42 |
| 8 A-C | 1000 | 51.7 (±0.3) | 72.0 (±0.8) | 150.0 (±0.7) | 546.2 (±10.0) | 969.8 (±31.8) |
| 8 D | 1000 | 51.7 (±0.3) | 46 | 43 | 42 | 42 |
| 9 A-C | 1000 | 51.7 (±0.3) | 73.6 (±1.2) | 151.7 (±2.8) | 594.8 (±19.7) | 995.6 (±9.3) |
| 9 D | 1000 | 51.7 (±0.3) | 46 | 43 | 44 | 44 |
| 10 A-C | 1000 | 51.7 (±0.3) | 72.3 (±0.7) | 150.5 (±2.9) | 621.7 (±37.4) | 1024.6 (±6.0) |
| 10 D | 1000 | 51.7 (±0.3) | 44 | 42 | 43 | 43 |
| 11 A-C | 1000 | 51.7 (±0.3) | 60.5 (±3.0) | 147.7 (±1.8) | 610.0 (±9.6) | 1002.2 (±14.9) |
| 11 D | 1000 | 51.7 (±0.3) | 43 | 40 | 56 | 52 |
| 12 A-C | 1000 | 51.7 (±0.3) | 70.7 (±0.3) | 150.0 (±3.4) | 620.0 (±33.2) | 998.4 (±6.1) |
| 12 D | 1000 | 51.7 (±0.3) | 44 | 42 | 42 | 40 |

Supplementary Table 2. Target and actual measured concentrations of I^-^ in cultures of N. maritimus SCM1 grown under a range of iodine and nitrogen ion concentrations; experiment number refers to conditions listed in table 1. Letters A-C in the experiment number refer to the biotic treatments, D refers to the abiotic control flasks.

| Experiment No | Target Concentration  (µM) | NH_4_^+^ concentration (µM) | Calculated I^-^Concentration following Inoculation (µM) | Measured Concentration (µM)  day 1 Mean (±Stdev) | Measured Concentration (µM)  day 3 Mean (±Stdev) | Measured Concentration (µM)  day 6 Mean (±Stdev) | Measured Concentration (µM)  day 8 Mean (±Stdev) |
| --- | --- | --- | --- | --- | --- | --- | --- |
| 1 A-C | 1000 | 1000 | 888.89 | 879.0 (±44.6) | 876.1 (±31.3) | 871.4 (±26.0) | 868.0 (±72.2) |
| 1 D |  | 1000 |  | 870 | 880 | 859 | 885 |
| 2 A-C | 1000 | 500 | 888.89 | 847.9 (±82.1) | 844.0 (±56.4) | 864.4 (±24.8) | 905.1 (±34.5) |
| 2 D |  | 500 |  | 876 | 701 | 897 | 919 |
| 3 A-C | 1000 | 100 | 888.89 | 817.3 (±67.6) | 863.1 (±80.6) | 888.5 (±17.6) | 874.8 (±23.7) |
| 3 D |  | 100 |  | 839 | 772 | 881 | 712 |
| 4 A-C | 500 | 500 | 444.44 | 424.6 (±52.5) | 447.6 (±17.7) | 416.9 (±56.8) | 454.4 (±10.4) |
| 4 D |  | 500 |  | 423 | 442 | 366 | 495 |
| 5 A-C | 100 | 100 | 88.89 | 87.5 (±4.9) | 86.4 (±8.7) | 89.5 (±2.1) | 89.3 (±2.8) |
| 5 D |  | 100 |  | 75 | 75 | 94 | 95 |
| 6 A-C | 100 | 1000 | 88.89 | 82.3 (±7.6) | 88.7 (±1.2) | 88.5 (±2.6) | 87.6 (±2.9) |
| 6 D |  | 1000 |  | 92 | 92 | 92 | 92 |
| 7 A-C | 10 | 1000 | 8.89 | 9.5 (±1.8) | 8.6 (±0.2) | 8.2 (±1.0) | 8.8 (±0.2) |
| 7 D |  | 1000 |  | 8.40 | 8.46 | 8.34 | 8.75 |
| 8 A-C | 1 | 1000 | 0.89 | 0.9 (±0.2) | 0.9 (±0.2) | 0.9 (±0.2) | 0.8 (±0.2) |
| 8 D |  | 1000 |  | 0.74 | 0.74 | 0.79 | 0.80 |
| 9 A-C | 0.1 | 1000 | 0.089 | 0.08 (±0.03) | 0.07 (±0.03) | 0.06 (±0.01) | 0.06 (±0.02) |
| 9 D |  | 1000 |  | 0.13 | 0.11 | 0.11 | 0.11 |
| 10 A-C | 0 | 1000 | 0 | BDL | BDL | BDL | BDL |
| 10 D |  | 1000 |  | BDL | BDL | BDL | BDL |
| 11 A-C | 0 | 1000 |  | 0.33 (±0.10) | 0.27 (±0.01) | 0.26 (±0.01) | 0.19 (±0.01) |
| 11 D |  | 1000 |  | 0.24 | 0.24 | 0.23 | 0.17 |
| 12 A-C | 0 | 1000 |  | BDL | BDL | 0.005 (±0.005) | 0.005 (±0.002) |
| 12 D |  | 1000 |  | BDL | BDL | 0.000 | 0.021 |

Supplementary Table 3. Target and actual measured concentrations of TII added to the biotic cultures during the Archaea growth experiment, alongside the actual measurements in the cultures following addition of the inoculum.

| Experiment No | Target I^-^Concentration  (µM) | Target IO_3_^-^Concentration (µM) | NH_4_^+^ concentration (µM) | Calculated TII Concentration following Inoculation (µM) | Measured Concentration (µM)  day 1 Mean (±Stdev) | Measured Concentration (µM)  day 3 Mean (±Stdev) | Measured Concentration (µM)  day 6 Mean (±Stdev) | Measured Concentration (µM)  day 8 Mean (±Stdev) |
| --- | --- | --- | --- | --- | --- | --- | --- | --- |
| 1 A-C | 1000 | 0 | 1000 | 888.89 | 862.4 (±22.7) | 876.2 (±22.6) | 866.9 (±35.9) | 857.5 (±62.7) |
| 1 D |  |  | 1000 |  | 846 | 871 | 931 | 890 |
| 2 A-C | 1000 | 0 | 500 | 888.89 | 846.1 (±53.5) | 846.9 (±54.7) | 865.0 (±30.1) | 894.6 (±15.0) |
| 2 D |  |  | 500 |  | 865 | 695 | 868 | 894 |
| 3 A-C | 1000 | 0 | 100 | 888.89 | 805.4 (±88.4) | 867.2 (±81.6) | 898.4 (±24.2) | 868.6 (±31.0) |
| 3 D |  |  | 100 |  | 828 | 741 | 880 | 691 |
| 4 A-C | 500 | 0 | 500 | 444.44 | 427.3 (±40.0) | 434.5 (±18.0) | 418.2 (±56.6) | 451.0 (±3.7) |
| 4 D |  |  | 500 |  | 420 | 469 | 367 | 453 |
| 5 A-C | 100 | 0 | 100 | 88.89 | 89.1 (±5.1) | 88.8 (±11.8) | 90.8 (±2.3) | 90.3 (±1.7) |
| 5 D |  |  | 100 |  | 76 | 78 | 96 | 95 |
| 6 A-C | 100 | 0 | 1000 | 88.89 | 84.6 (±7.1) | 90.9 (±2.4) | 90.7 (±1.4) | 90.2 (±2.4) |
| 6 D |  |  | 1000 |  | 93 | 93 | 95 | 95 |
| 7 A-C | 10 | 0 | 1000 | 8.89 | 8.8 (0.2) | 9.1 (±0.2) | 8.3 (±0.9) | 9.2 (±0.1) |
| 7 D |  |  | 1000 |  | 8.64 | 8.70 | 8.61 | 8.98 |
| 8 A-C | 1 | 0 | 1000 | 0.89 | 0.83 (±0.17) | 0.85 (±0.19) | 0.89 (±0.19) | 0.85 (±0.17) |
| 8 D |  |  | 1000 |  | 0.77 | 0.79 | 0.84 | 0.83 |
| 9 A-C | 0.1 | 0 | 1000 | 0.089 | 0.08 (±0.02) | 0.06 (±0.03) | 0.06 (±0.01) | 0.05 (±0.02) |
| 9 D |  |  | 1000 |  | 0.11 | 0.11 | 0.11 | 0.11 |
| 10 A-C | 0 | 0 | 1000 | 0 | BDL | BDL | BDL | BDL |
| 10 D |  |  | 1000 |  | BDL | BDL | BDL | BDL |
| 11 A-C | 0 | 1000 | 1000 | 888.89 | 885.3 (±5.1) | 828.8 (±109.1) | 898.1 (±14.8) | 936.4 (±12.0) |
| 11 D |  |  | 1000 |  | 916.97 |  | 819.40 | 944.54 |
| 12 A-C | 0 | 1 | 1000 | 0.89 | 0.9 (± 0.02) | 0.9 (±0.06) | 0.8 (±0.1) | N/A |
| 12 D |  |  | 1000 |  | 0.82 | 0.89 | 0.87 | N/A |
